# Comparative study on the efficacy of Conbercept and Aflibercept in the treatment of neovascular age-related macular degeneration

**DOI:** 10.1038/s41598-024-62536-8

**Published:** 2024-05-25

**Authors:** Hao Xie, Huan Ju, Jing Lu, Xing Wang, Hui Peng

**Affiliations:** 1 Department of Ophthalmology, the First Affiliated Hospital of Chongqing Medical University, No.1, Youyi Road, Chongqing, China; 2 Department of Clinical Medicine, Chongqing Medical University, No.1, Yixueyuan Road, Chongqing, China

**Keywords:** Neovascular age-related macular degeneration, Receptor fusion proteins drugs, Efficacy comparison, Conbercept, Aflibercept, Diseases, Medical research

## Abstract

This study compares the effectiveness of Conbercept and Aflibercept in treating neovascular age-related macular degeneration (nAMD). Conducted at the First Affiliated Hospital of Chongqing Medical University's Ophthalmology Department (May 2020–May 2023), this prospective study enrolled 159 nAMD patients. Participants were randomly divided into two groups: one receiving 0.5 mg Conbercept and the other 2 mg Aflibercept intravitreal injections. Over 12 months, the study, employing a Treat-and-Extend (T&E) regimen, assessed Best-Corrected Visual Acuity (BCVA), Central Retinal Thickness (CRT) changes and injection frequency. Of the 159 patients, 137 (149 eyes) completed the study. No significant age difference was found between the groups (P = 0.331). After 12 months, BCVA improved similarly in both groups (Conbercept: 52.8 ± 18.9, Aflibercept: 52.0 ± 19.7 letters; P = 0.820). CRT reduction was also comparable (Conbercept: 246.3 ± 82.8 µm, Aflibercept: 275.9 ± 114.3 µm; P = 0.079). Injection frequencies averaged 6.9 ± 0.7 (Conbercept) and 6.7 ± 0.7 (Aflibercept; P = 0.255). Subtype analysis revealed Type 1 MNV had higher baseline BCVA and lower CRT, with more frequent injections compared to other types. Both Conbercept and Aflibercept are clinically similar in efficacy for nAMD, with the T&E regimen proving therapeutically effective and potentially reducing patient costs. Anti-VEGF treatment efficacy varies across nAMD subtypes, indicating a potential benefit in tailored treatments for specific subtypes.

*Clinical trial registration number* NCT05539235 (Protocol Registration and Results System).

## Introduction

Macular degeneration, the primary cause of severe, irreversible vision loss in those over 55, is commonly termed age-related macular degeneration (AMD)^1^. It manifests in two forms: dry AMD and the more rapidly vision-impairing wet AMD. In wet AMD, accelerated by vascular endothelial growth factor (VEGF) and placental growth factor (PLGF), choroidal neovascularization (CNV) occurs in the macula. This leads to retinal exudation, hemorrhage, and ultimately central vision loss^2^, hence its alternative name, neovascular AMD (nAMD).

In clinical practice, nAMD diagnosis integrates fundoscopic exams with imaging techniques like fluorescein fundus angiography (FFA) and indocyanine green angiography (ICGA), previously gold standards. Recent high-resolution tomography, including optical coherence tomography (OCT) and OCT angiography (OCTA), offers detailed, three-dimensional views of the retinal and choroidal vasculature^3^, revealing new vessel size, shape, and location.Based on the classification of the anatomical levels of neovascularization, the International Consensus on Naming for Age Related Macular Degeneration Research Group has formed a consensus on the naming of nAMD^4^. The consensus suggests using macular neovascularization (MNV) instead of CNV, and categorizing nAMD into three types based on the anatomical location of MNV: Type 1 (MNV between the retinal pigment epithelium and Bruch membrane, including polypoidal choroidal vasculopathy), Type 2 (MNV from the choroidal capillaries, growing under the retinal nerve fiber layer), and Type 3 (MNV from the retina, extending to the outer retina and potentially leading to pigment epithelial detachment and CNV). Besides these, mixed-type lesions exhibit features of multiple types."

Internationally, nAMD treatments include drug therapy, surgical options, and photodynamic therapy, with anti-VEGF drug therapy as the recommended first-line approach^5^. This category mainly involves receptor fusion proteins like Conbercept and Aflibercept.

Conbercept, developed in China, is a recombinant fusion protein with high affinity for all VEGF-A, VEGF-B, VEGF-C, PLGF subtypes, and a longer half-life^6^. It uniquely includes VEGFR-2's domain 4, enhancing its efficacy^7,8^, and has shown 50 times higher VEGF affinity than bevacizumab^9^. In the PHOENIX trial^10^, Conbercept improved nAMD visual outcomes with less frequent dosing.

Aflibercept, a fully humanized fusion protein combining VEGFR-1 and -2 domains with IgG1 Fc fragment, binds to all VEGF-A, VEGF-B, PLGF subtypes^11^, offering superior affinity and half-life over monoclonal antibodies.Its bi-monthly administration matches the effectiveness of monthly ranibizumab in maintaining visual acuity in new nAMD cases^12^. Despite studies comparing monoclonal antibodies and receptor fusion proteins^13–16^, there's limited research directly comparing receptor fusion proteins like Conbercept and Aflibercept in nAMD. This study addresses this gap, evaluating their efficacy differences using a Treatment and Extend (T&E) regimen in nAMD."

## Methods

### Study design

This 12-month, randomized, phase IV trial at The First Affiliated Hospital of Chongqing Medical University compared the efficacy of 0.5mg Conbercept and 2mg Aflibercept in treating nAMD. Participants were equally randomized to each treatment group. Written informed consent was secured before examinations. The study adhered to the Declaration of Helsinki and received approval from the Institutional Review Board of Chongqing Medical University, the First Affiliated Hospital (Approval No. 20199401).

### Sample size determination

This study is a randomized controlled trial comparing the efficacy of Conbercept group versus Aflibercept group. The primary outcome measure is the change in Best Corrected Visual Acuity (BCVA) among the subjects. Based on the preliminary experiment results at the sixth month, the mean change in BCVA for the Conbercept group was 5.6 ± 5.85 letters, and for the Aflibercept group, it was 1.2 ± 8.16 letters. Setting a two-sided α at 0.05 and a power of 90%, the sample size was calculated using the following formula:

$$n = \frac{{2\left( {z_{\alpha } + z_{\beta } } \right)^{2} * \sigma^{2} }}{{\delta^{2} }}.$$

As a result, n = 55 cases per group were determined. Considering a 1:1 randomization for treatment and control groups, each group requires 55 subjects. Accounting for a 15% dropout and refusal rate, each group should ultimately include at least 65 participants, totaling a minimum of 130 subjects for the study.

### Examination and enrolment

In this prospective study at the First Affiliated Hospital of Chongqing Medical University (May 2020 to May 2023), patients were randomly assigned to either the Conbercept or Aflibercept group. Inclusion criteria were age ≥ 50 years and confirmed nAMD via mydriatic slit lamp exam, OCT, OCTA, FFA, and ICGA, with no prior nAMD treatments. Only patients able to follow up for at least 12 months were included. Exclusion criteria encompassed coexisting fundus diseases (like retinal detachment, pathological myopia, proliferative diabetic retinopathy, glaucoma, retinal vascular diseases, macular membrane, macular hole), past internal eye surgery or fundus laser treatment, severe ocular trauma history, significant refractive medium clouding hindering fundus observation, and inability to maintain regular treatment follow-up or follow-up less than 12 months.

*BCVA* BCVA was recorded for all eligible patients pre-treatment, and at 1, 3, 6 months, and 1 year post-treatment, using the ETDRS chart for final assessment.

*OCT and OCTA* Examinations utilized the CIRRUS HD-OCT 5000 with pupil dilation to approximately 7mm.

*FFA and ICGA* Examinations used the Heidelberg Spectralis HRA angiography system from Germany.

### Treatment

After routine preoperative exams and assessments, informed consent was obtained from patients and their families. Both groups underwent standardized intravitreal drug injections. According to different groups, the patients were given intravitreal injection of Conbercept (0.5mg) or Aflibercept ( 2 mg).

The patients in both groups adopted the T&E protocol^17^, namely, the initial treatment stage was three load injections, Conbercept (0. 5mg) or Aflibercept (2mg) was injected once every four weeks for three times, and the treatment interval was extended to eight weeks after that. Following the fourth injection, the subsequent treatment interval will be determined based on the patient's BCVA, CRT, OCT, and fundus photography. The inter-treatment interval will not be shorter than 8 weeks and shall not exceed 16 weeks. Treatment interval extension: an extension of the treatment interval is warranted when the following conditions are concurrently satisfied: (1) Vision stability with a < 5-letter decrease in sequential BCVA assessments; (2) Retinal thickness stability, with CRT increment of < 50μm; (3) Absence of persistent intraretinal fluid (IRF) and subretinal fluid (SRF), or stable persistent retinal fluid over three consecutive visits, attributed to long-standing anatomical changes or fibrosis without active CNV; (4) No new neovascularization; and (5) No new macular hemorrhage. Treatment interval retention: the established interval may be upheld when all specified parameters are met: (1) Visual acuity decline of < 5 letters; (2) OCT imaging reveals no new retinal fluid accumulation or a decrease in fluid volume relative to the last visit; (3) Absence of new neovascularization; and (4) No new macular hemorrhage. Treatment interval reduction: treatment intervals should be decreased upon the occurrence of any of the following conditions: (1) A reduction in visual acuity of ≥ 5 letters (or 1 line); (2) Recurrent or persistent retinal fluid associated with a decrease in vision; (3) Development of new neovascularization; (4) Onset of new macular hemorrhage; (5) Continued activity of the lesion following an attempt to extend the treatment interval.

### Follow-up

Outpatient follow-ups recorded BCVA, CRT, intraocular pressure, and total anti-VEGF treatments at 12 months, at baseline, and at 1, 3, 6, and 12 months of treatment. Fundus lesions were monitored before and after treatment. MNV classification was based on baseline OCTA, with neovascular regression assessed at each follow-up. The study also observed ocular and systemic complications related to drug injections, including subconjunctival hemorrhage, endophthalmitis, vitreous hemorrhage, and retinal detachment.

### Masking

Researcher A logged into the central randomization system to obtain a random number, and finally formed a random allocation table. They were randomly divided into the Conbercept group and the Aflibercept group in a 1:1 ratio without blinding. Doctor B consistently measured and recorded all patients' BCVA. Additionally, Doctor C from the Photographic Reading Centre evaluated and classified all OCT, OCTA, FFA, and ICGA images. Both Doctors B and C, as well as the patients, remained unaware of the group allocations.

### Statistical analyses

Data from both groups were analyzed using SPSS22.0. The independent sample t-test was applied to measurement data, presented as mean ± standard deviation. Enumeration data were evaluated using the Chi-square test and expressed as proportions. A P-value < 0.05 was considered statistically significant.

### Ethical approval and consent to participate

The Ethics Committee of Clinical Research of The First Affiliated Hospital of Chongqing Medical University approved and reviewed the studies involving human participants(Grant number, 20190401). The patients/participants provided their written informed consent to participate in this study.

## Consent for publication

The patient’s written and informed consent was obtained.

## Results

The study enrolled 159 patients (May 2020 to May 2023), including 79 patients in the Conbercept group and 80 patients in the Aflibercept group, with 137 (149 eyes) completing the 12-month follow-up, 82 eyes received Conbercept and 67 Aflibercept. 9 participants in the Conbercept group and 13 in the Aflibercept group voluntarily withdrew from the study, citing personal reasons. Baseline characteristics (age, gender, BCVA, CRT, OCTA MNV classification) showed no significant differences between groups, detailed in Table 1.

**Table 1:** Baseline characteristics.

Variables	Conbercept (N = 82)	Aflibercept (N = 67)	*P* value
Age (years)			
Mean ± SD	71.0 ± 9.4	72.3 ± 7.8	0.331
Median (range)	70.5 (51.0–92.0)	71.0 (53.0–91.0)	
Sex, No. (%)			0.423
Male	47 (57.3)	34 (50.7)	
Female	35 (42.7)	33 (49.3)	
Frequency of injections			0.255
Mean ± SD	6.8 ± 0.7	6.7 ± 0.7	
Median (range)	7.0 (6.0–8.0)	7.0 (6.0–8.0)	
BCVA baseline (letters)			
Mean ± SD	48.6 ± 18.3	46.8 ± 14.7	0.508
Median (range)	51.5 (18.0–81.0)	45.0 (19.0–78.0)	
CRT baseline (μm)			
Mean ± SD	378.9 ± 134.6	403.2 ± 144.0	0.288
Median (range)	353.5 (140.3–840.9)	392.6 (145.1–804.1)	
MNV classification, No. (%)			0.452
1	33 (40.2)	19 (28.4)	
2	38 (46.3)	39 (58.2)	
3	7 (8.5)	5 (7.5)	
Mixed	4 (4.9)	4 (6.0)	
RPE atrophy baseline, No. (%)			0.395
Yes	16 (19.5)	17 (25.4)	
No	66 (80.5)	50 (74.6)	

SD, standard deviation; BCVA, best-corrected visual acuity; CRT, central retinal thickness; MNV, macular neovascularization.

### BCVA

**Figure 1:**
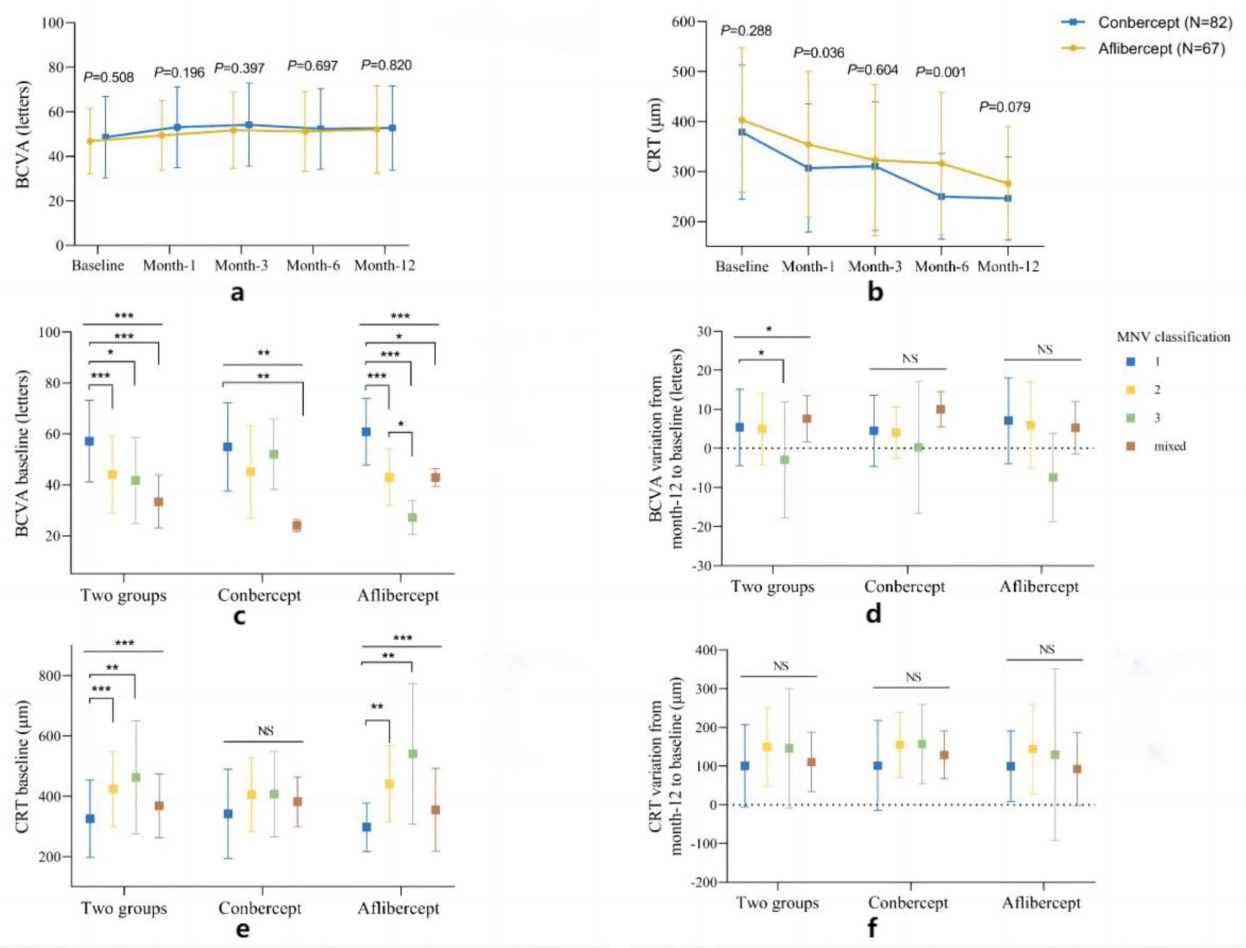
(**a**) Mean BCVA (letter Score) at baseline and at each follow-up time until month 12 in the two groups; (**b**) Mean CRT (μm) at baseline and at each follow-up time until month 12 in the two group; (**c**) BCVA baseline (letter Score) of four types of MNV; (**d**) BCVA variation from month-12 to baseline (letter Score) of four types of MNV; (**e**) CRT baseline of four types of MNV; f:CRT variation from month-12 to baseline (letter Score) of four types of MNV. “**” means P < 0.01, “***” means P < 0.001.

**Figure 2:**
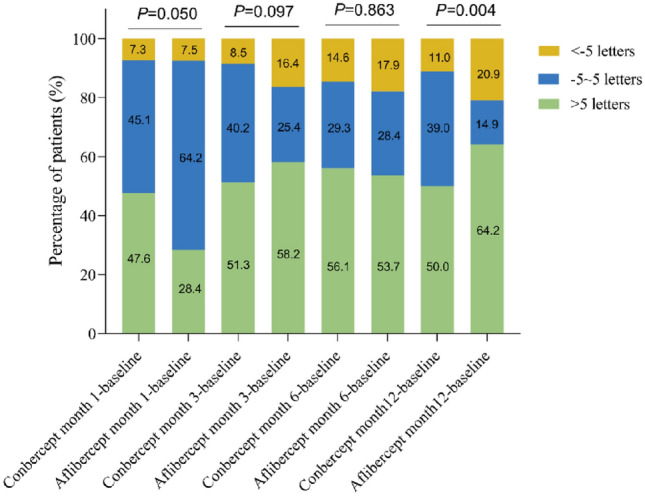
Classifying according to the change in BCVA letter score.

At 12 months, no significant differences in BCVA or mean changes were observed between groups, detailed data are shown in Fig. 1a and Table 2. 50.0% of Conbercept and 64.2% of Aflibercept patients showed > 5 letter BCVA improvement (Fig. 2). Type 1 MNV had a higher BCVA baseline (57.1 ± 16.0 letters) than other types (P < 0.05). At 12 months, no significant differences in mean changes were observed between four types, detailed data are shown in Table 3. After grouping all patients according to MNV subtype, an analysis was conducted on baseline BCVA and changes in BCVA (Fig. 1c, Fig. 1d, Table 3).

**Table 2:** Comparison the difference of BCVA letter score and CRT between the two groups.

Variables	Conbercept (N = 82)	Aflibercept (N = 67)	*P* value
Month-1			
BCVA (letters)			
Mean ± SD	53.1 ± 18.1	49.5 ± 15.7	0.196
Median (range)	56.5 (20.0–82.0)	47.0 (20.0–83.0)	
BCVA variation from baseline (letters)			
Mean ± SD	4.5 ± 7.4	2.7 ± 5.5	0.098
Median (range)	4.0 (−21.0 to 30.0)	3.0 (−10.0 to 19.0)	
CRT (μm)			
Mean ± SD	306.9 ± 128.2	354.3 ± 145.6	**0.036**
Median (range)	263.0 (113.4–688.7)	325.1 (136.6–773.3)	
CRT variation from baseline (μm)			
Mean ± SD	72.0 ± 81.3	48.9 ± 51.4	**0.037**
Median (range)	77.5 (−114.1 to 236.5)	49.4 (−48.2 to 163.6)	
Month-3			
BCVA (letters)			
Mean ± SD	54.2 ± 18.7	51.7 ± 17.2	0.397
Median (range)	58.0 (18.0–84.0)	53.0 (12.0–83.0)	
BCVA variation from baseline (letters)			
Mean ± SD	5.7 ± 7.8	5.0 ± 9.0	0.611
Median (range)	5.0 (−17.0 to 36.0)	7.0 (−16.0 to 29.0)	
CRT (μm)			
Mean ± SD	310.8 ± 128.2	322.7 ± 151.6	0.604
Median (range)	292.9 (98.1–832.7)	292.8 (107.3–728.6)	
CRT variation from baseline (μm)			
Mean ± SD	68.1 ± 94.2	80.6 ± 89.7	0.413
Median (range)	71.6 (−193.7 to 332.9)	79.5 (−100.8 to 247.2)	
Month-6			
BCVA (letters)			
Mean ± SD	52.4 ± 18.1	51.2 ± 17.9	0.697
Median (range)	49.0 (18.0–83.0)	51.0 (13.0–83.0)	
BCVA variation from baseline (letters)			
Mean ± SD	3.8 ± 9.7	4.4 ± 9.8	0.691
Median (range)	6.0 (−25.0 to 35.0)	6.0 (−18.0 to 27.0)	
CRT (μm)			
Mean ± SD	250.0 ± 85.7	316.1 ± 142.5	**0.001**
Median (range)	224.5 (123.0–568.4)	285.2 (126.0–736.1)	
CRT variation from baseline (μm)			
Mean ± SD	128.9 ± 100.6	87.1 ± 83.8	**0.007**
Median (range)	136.6 (−121.5 to 336.5)	88.5 (−88.3 to 255.4)	
Month-12			
BCVA (letters)			
Mean ± SD	52.8 ± 18.9	52.0 ± 19.7	0.820
Median (range)	53.0 (16.0–85.0)	51.0 (10.0–85.0)	
BCVA variation from baseline (letters)			
Mean ± SD	4.2 ± 8.8	5.3 ± 11.2	0.515
Median (range)	4.5 (−25.0 to 24.0)	7.0 (−20.0 to 31.0)	
CRT (μm)			
Mean ± SD	246.3 ± 82.8	275.9 ± 114.3	0.079
Median (range)	227.1 (85.7–520.4)	273.0 (100.2–673.1)	
CRT variation from baseline (μm)			
Mean ± SD	132.5 ± 100.7	127.3 ± 117.5	0.772
Median (range)	147.9 (−120.6 to 403.2)	132.9 (−142.5 to 483.8)	

BCVA, best-corrected visual acuity; SD, standard deviation. CRT, central retinal thickness.

Significant values are in bold.

**Table 3:** Group analysis based on MNV classification.

Variables	Overall	1	2	3	Mixed	*P* value
Conbercept	82	33	38	7	4	
Frequency of injections, Mean ± SD	6.8 ± 0.7	7.2 ± 0.6*^,&^	6.4 ± 0.6^$^	7.1 ± 0.9	6.3 ± 0.5	** < 0.001**
BCVA baseline (letters), Mean ± SD	48.6 ± 18.3	54.9 ± 17.3^&^	45.1 ± 18.1	52.0 ± 13.9	24.0 ± 2.4	**0.004**
CRT baseline (μm), Mean ± SD	378.9 ± 134.6	341.6 ± 147.7	405.6 ± 122.0	407.2 ± 141.1	382.1 ± 82.6	0.229
Month-12						
BCVA variation from baseline (letters), Mean ± SD	4.2 ± 8.8	4.5 ± 9.1	4.0 ± 6.6	0.3 ± 16.9	10.0 ± 4.5	0.371
CRT variation from baseline (μm), Mean ± SD	132.5 ± 100.7	101.7 ± 116.2	155.1 ± 83.8	157.3 ± 102.4	129.1 ± 61.5	0.143
Aflibercept	67	19	39	5	4	
Frequency of injections, Mean ± SD	6.7 ± 0.7	7.3 ± 0.7*	6.3 ± 0.5^$^	7.2 ± 0.8	7.0 ± 0.8	** < 0.001**
BCVA baseline (letters), Mean ± SD	46.8 ± 14.7	60.8 ± 13.1*^,#,&^	42.9 ± 11.1^$^	27.2 ± 6.7	42.8 ± 3.5	** < 0.001**
CRT baseline (μm), Mean ± SD	403.2 ± 144.0	297.6 ± 80.2*^,#^	442.0 ± 126.1	540.4 ± 232.9	355.2 ± 137.2	** < 0.001**
Month-12						
BCVA variation from baseline (letters), Mean ± SD	5.3 ± 11.2	7.1 ± 11.0	6.0 ± 11.0	−7.4 ± 11.3	5.3 ± 6.7	0.067
CRT variation from baseline (μm), Mean ± SD	127.3 ± 117.5	99.6 ± 91.0	144.1 ± 115.7	129.6 ± 221.7	92.4 ± 94.5	0.540
Two groups	149	52	77	12	8	
Frequency of injections, Mean ± SD	6.7 ± 0.7	7.2 ± 0.6*	6.4 ± 0.5^$^	7.2 ± 0.8	6.6 ± 0.7	** < 0.001**
BCVA baseline (letters), Mean ± SD	47.8 ± 16.8	57.1 ± 16.0*^, #, $^	44.0 ± 14.9	41.7 ± 16.9	33.4 ± 10.4	** < 0.001**
CRT baseline (μm), Mean ± SD	389.8 ± 138.9	325.6 ± 128.1*^, #^	424.1 ± 124.7	462.7 ± 187.9	368.7 ± 105.8	** < 0.001**
Month-12						
BCVA variation from baseline (letters), Mean ± SD	4.7 ± 9.9	5.4 ± 9.8^#^	5.0 ± 9.1	−2.9 ± 14.8	7.6 ± 5.9	**0.042**
CRT variation from baseline (μm), Mean ± SD	130.2 ± 108.2	101.0 ± 106.8	149.5 ± 100.7	145.8 ± 154.3	110.7 ± 76.4	0.079

Boldface represented *P* value < 0.05. * represented *P* value < 0.05 between MNV 1 and MNV 2; # represented *P* value < 0.05 between MNV 1 and MNV 3; & represented *P* value < 0.05 between MNV 1 and MNV 4; $ represented *P* value < 0.05 between MNV 2 and MNV 3; MNV, macular neovascularization; BCVA, best-corrected visual acuity; SD, standard deviation; CRT, central retinal thickness.

At month-12, the proportion of patients with stable or improved vision in Conbercept group is higher than Aflibercept group, while the proportion of patients with reduced vision is lower than that Aflibercept group (Fig. 2).

### CRT

During the 12-month treatment period, both groups of patients exhibited a continuous decrease in CRT compared to baseline levels (Fig. 1b). At the 1st and 6th months, the Conbercept group showed a significantly greater reduction in CRT compared to the Aflibercept group, as indicated in Table 3. After grouping all patients according to MNV subtype, an analysis was conducted on CRT baseline and changes in CRT (Fig. 1e,f, Table 3). At month-12 the CRT baseline for Type 1 MNV was 325.6 ± 128.1 µm, significantly lower than that of other types of MNV (P < 0.05)(Table 3).

### Injection times

**Figure 3:**
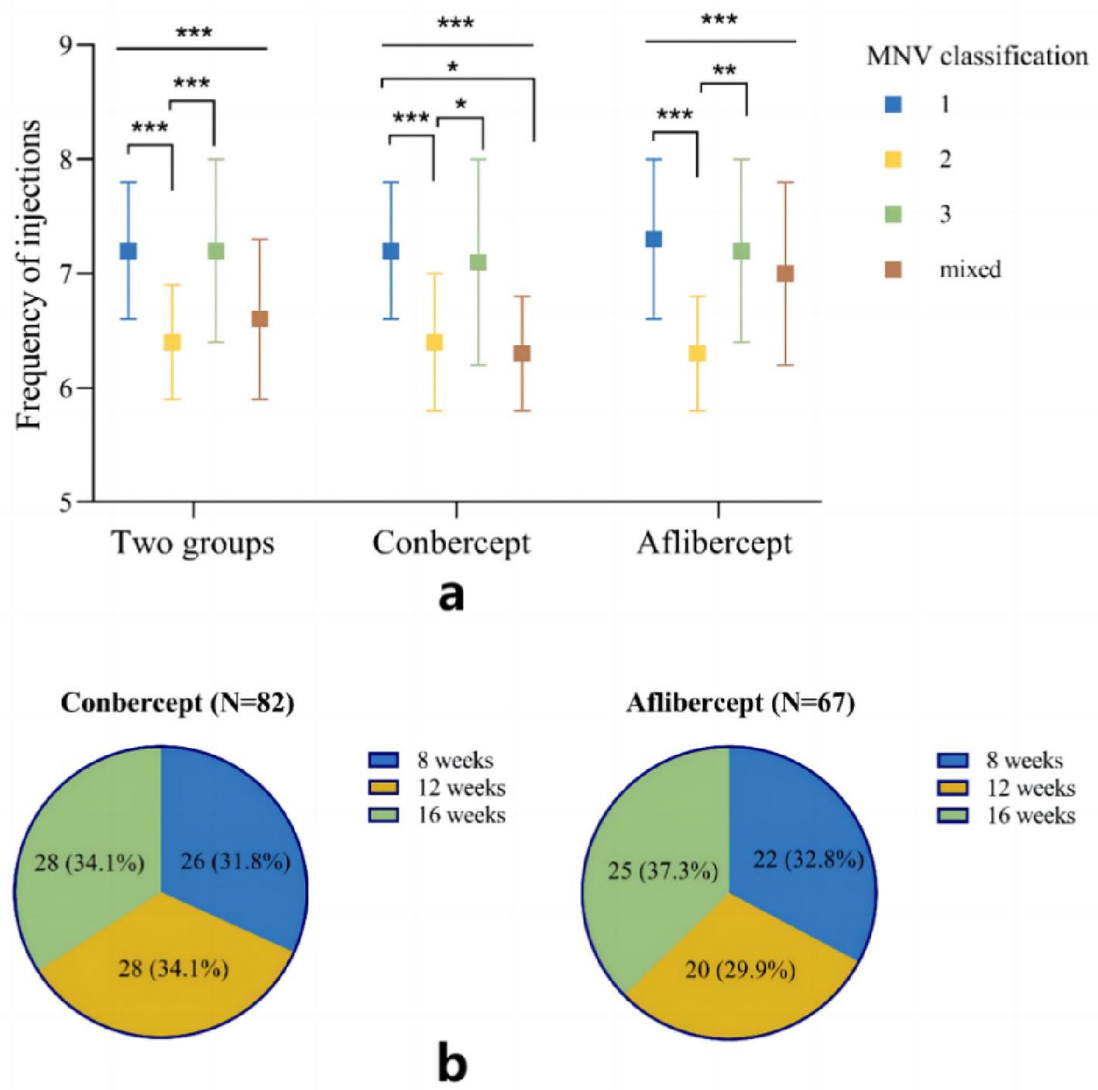
(**a**) Frequency of injections of four types of MNV; (**b**) The last injection interval before Month-12. “**” means P < 0.01, “***” means P < 0.001.

At the 12-month follow-up, the average number of intravitreal injections in the Conbercept group and the Aflibercept group, respectively, with no statistically significant difference between the two groups (p = 0.255). Detailed data of the intervals for the last injection are shown in Fig. 3b.

Over the course of 52 weeks (12 months), the distribution of injection frequencies for 6, 7, and 8 injections in the Conbercept group was 30 people (36.59%), 39 people (47.56%), and 13 people (15.85%), respectively, while in the Aflibercept group, it was 32 people (47.76%), 25 people (37.31%), and 10 people (14.93%). There was no statistically significant difference between the groups in individuals who received 6 injections (P = 0.36).

Injection frequency across various subtypes was systematically analyzed. The mean injection count for Type 1 was 7.2 ± 0.6, significantly surpassing Type 2's 6.4 ± 0.5 and the mixed type's 6.6 ± 0.7 (P < 0.05). Notably, Type 3's mean injection count stood at 7.2 ± 0.8, exceeding that of Type 2 (P < 0.05), as detailed in Fig. 3a and Table 3.

### Postoperative complications

In this study, a total of 5 cases of subconjunctival hemorrhage were observed (4 cases in the Conbercept group and 1 case in the Aflibercept group). These cases did not receive any specific treatment, and the symptoms resolved spontaneously after observation. There were no postoperative complications such as elevated intraocular pressure, vitreous hemorrhage, intraocular inflammation, or retinal detachment observed in either group of patients.

## Discussion

This study, the first prospective analysis over a year comparing Conbercept and Aflibercept in a real-world context, reveals their similar efficacy and safety for nAMD. With no significant differences in injection frequency or safety, both are viable nAMD treatment options.

Conbercept, a 143 kDa fusion protein, combines VEGFR1Ig2, VEGFR2Ig3, and VEGFR2Ig4 of human VEGF receptors with IgG1 Fc^10^, binding all VEGFA, VEGF-B, VEGF-C, and PlGF subtypes^18,19^. Aflibercept, at 115 kDa, fuses VEGFR1Ig2 and VEGFR2Ig3 with IgG1 Fc^20,21^, targeting VEGF-A, VEGF-B, and PlGF ^22^.In rabbit vitreous, Conbercept has a 4.2-day half-life, longer than Aflibercept's 3.63 days^23,24^. Conbercept's unique inclusion of VEGFR2's fourth domain enhances VEGF affinity and lowers extracellular matrix adhesion, potentially boosting its anti-angiogenic effectiveness^9^. Despite these differences, comparative studies in nAMD show no clear superiority of either drug in improving visual acuity, anatomical outcomes, or treatment frequency.In studies utilizing the 3 + T&E regimens (injection intervals extended or shortened by 4 weeks), the COCOA study, a pioneering multicentric prospective Chinese trial, compared Conbercept 3 + T&E (treatment intervals adjusted by 4 weeks) against a 3 + PRN regimen in 501 patients. By week 48, more than half of the participants had received 6 or fewer injections. In the 3 + T&E cohort, visual acuity improved by an average of 8.6 letters, with about 62% of patients having ≥ 12-week intervals and 42% extending to 16 weeks^20^.The ALTAIR Study, administering 2 mg Aflibercept intravitreally, observed an average improvement of 8.4 letters in visual acuity at 52 weeks and an average of 6.9 injections over 12 months, accompanied by a mean CRT reduction of 126.1 μm. Approximately 49.6% of the patients in this study extended the final injection interval to ≥ 12 weeks, and 40.7% to 16 weeks ^25^. Moreover, in both AURORA and PHOENIX studies assessing Conbercept and the VIEW1 and VIEW2 studies evaluating Aflibercept, the comparative assessment in terms of BCVA improvement, CRT reduction, and injection frequency did not demonstrate clear dominance of either treatment^10,26,27^.

Drug clearance from the vitreous hinges on molecular size. Due to their larger molecular weight, these drugs minimally penetrate the blood-ocular barrier, ensuring high selectivity and few systemic adverse reactions, confirming their safety. This study finds no significant difference between the two drugs in efficacy, injection frequency, or adverse reactions, consistent with previous research. While Conbercept's additional structural domain might suggest greater efficacy, this doesn't necessarily translate to clinical significance.As AMD is influenced by various factors, patients' responses to anti-VEGF treatments can vary, with some experiencing reduced effectiveness over time.Notably, despite these treatments, AMD-related blindness incidences continue to increase annually^28^.

A U.S. study of 98,821 AMD eyes undergoing intravitreal anti-VEGF therapy showed a general visual acuity decline over four years^29^. This decrease is partly due to these treatments' inability to address factors like age, genetics, and environmental and cardiovascular influences. AMD's Geographic Atrophy (GA) grows about 0.33 mm/year, with a 38% incidence rate five years after starting anti-VEGF therapy^30^. Normally, ocular VEGF is essential for RPE survival and choroidal endothelial cell function^31^, so anti-VEGF treatments might increase GA risk^32^, possibly diminishing or even worsening visual acuity over time. This could explain the converging efficacy in long-term therapy. However, studies suggest no direct link between anti-VEGF dosing or injection frequency and macular atrophy rates in nAMD^33^.

After a year, visual acuity gains in both groups were 4.18 ± 8.80 and 5.25 ± 11.23 letters, lower than previous CATT study results.

Contributing factors include lower baseline visual acuity (Conbercept: 48.59 ± 18.33, Aflibercept: 46.79 ± 14.70 letters) compared to the 2012–2015 average of 53.1 letters^29^. This suggests more advanced disease at treatment onset, typically offering limited visual improvement.Also, about 46% of patients had Type 2 MNV, which, despite initial responsiveness to treatment, often leads to fibrotic scarring, potentially impairing long-term vision^34^. The high proportion of Type 2 cases may limit overall BCVA improvement. Furthermore, the T&E regimen in this study, with 4-week extension and shortening intervals (minimum 8 weeks, maximum 16 weeks), lacked adaptability for patients needing more frequent treatments, potentially leading to lesser visual improvement and not fully addressing specific needs. The fixed interval scheme could explain why more patients extended their last treatment interval to ≥ 12 weeks compared to prior studies.

In a subtype-based analysis, Type 1 neovascularization patients, with higher initial BCVA, needed more frequent injections but didn't show significant visual improvement over other types. Typically, Type 1 patients have higher baseline BCVA and better vision improvement, requiring more treatments^35^. This MNV, located beneath the RPE, preserves visual acuity by not damaging the RPE and maintaining its barrier and transport functions, reflected in this study's lower average CRT for Type 1. However, the deeper neovascularization location reduces the concentration of anti-VEGF medication reaching it to just 11.9% of that in the vitreous cavity^23^. Consequently, maintaining anti-VEGF treatment efficacy over time is challenging, and patients with this type often experience recurrent episodes, needing more injections for effective management.

In this study, Type 2 neovascularization incidence (51.7%) was higher than in past CATT studies (19.2–23.7%)^36^. This could reflect regional differences due to the study's single-center nature. Type 2 patients had lower baseline BCVA but needed fewer treatments, and their visual improvement was not significantly inferior to other subtypes.

Type 2 MNV, penetrating and located above the RPE, often presents with poorer vision.

Yet, its accessibility to anti-VEGF medications leads to better treatment response and fewer injections^37^, however, there are studies indicating that Type 2 neovascularization may require a higher frequency of treatment interventions^35^. This increased treatment frequency in Type 2 patients could be attributed to their higher susceptibility to fibrotic scarring^38^, it can impede improvements in visual acuity and the efficacy of treatment, potentially leading to more frequent injections in clinical practice. The prevalence of Type 2 in our study may have influenced average baseline BCVA, BCVA improvement, CRT reduction, and injection numbers. Type 3 patients, typically with poorer baseline vision, required more frequent injections, and experienced BCVA decline by 12 months. This subtype is characterized by significant RPE damage, with 6 patients (50%) already exhibiting atrophic changes in the macular RPE at the onset of treatment. By the 12th month, 2 more patients developed macular RPE atrophy. Previous studies have also indicated a higher propensity for Geographic Atrophy (GA) in patients with Type 3 neovascularization^30^. Mixed subtype patients had the poorest baseline BCVA and needed fewer injections, though the small sample size limits generalization.Considering these subtype differences, tailored treatments are essential, especially for unstable types like 1 and 3, where shorter interval dosing might be more effective. Future therapies should thus be subtype-specific for nAMD.

## Limitations

This single-center study may limit the generalizability of population characteristics. Its small sample size, especially for Type 3 and mixed subtypes, could bias data analysis. The one-year follow-up might be insufficient for comparing long-term drug efficacy. Fixed treatment intervals, not tailored to patient conditions, may have affected anti-VEGF therapy effectiveness. Additionally, many patients had poor baseline visual acuity, suggesting advanced disease stages, which could influence OCT and OCTA subtype-based observations and outcomes.

## Conclusion

Clinically, the efficacy of the two drugs may be similar. The T&E regimen, effective therapeutically, could also lessen patient economic burden. Responsiveness to anti-VEGF treatments differs among nAMD subtypes, suggesting future treatments should be customized by subtype for optimal patient benefit.

## Data Availability

The data underlying this article are available in the article and in its online supplementary material.
